# Strategy for eliciting antigen-specific CD8+ T cell-mediated immune response against a cryptic CTL epitope of merkel cell polyomavirus large T antigen

**DOI:** 10.1186/2045-3701-2-36

**Published:** 2012-10-24

**Authors:** Bianca P Gomez, Connie Wang, Raphael P Viscidi, Shiwen Peng, Liangmei He, T-C Wu, Chien-Fu Hung

**Affiliations:** 1 Departments of Pathology, Johns Hopkins Medical Institutions, Baltimore, MD, USA; 2 Pediatrics, Johns Hopkins Medical Institutions, Baltimore, Maryland, USA; 3 Obstetrics and Gynecology, Johns Hopkins Medical Institutions, Baltimore, MD, USA; 4 Molecular Microbiology and Immunology, Johns Hopkins Medical Institutions, Baltimore, MD, USA; 5 Oncology, Johns Hopkins Medical Institutions, Baltimore, MD, USA; 6 Departments of Pathology and Oncology, The Johns Hopkins University School of Medicine, CRB II Room 307, 1550 Orleans Street, Baltimore, MD, 21231, USA

**Keywords:** DNA vaccine, Gene therapy, Merkel cell polyomavirus, Cryptic CTL Epitope, Large T antigen

## Abstract

**Background:**

Merkel cell carcinoma (MCC) is a relatively new addition to the expanding category of oncovirus-induced cancers. Although still comparably rare, the number of cases has risen dramatically in recent years. Further complicating this trend is that MCC is an extremely aggressive neoplasm with poor patient prognosis and limited treatment options for advanced disease. The causative agent of MCC has been identified as the merkel cell polyomavirus (MCPyV). The MCPyV-encoded large T (LT) antigen is an oncoprotein that is theorized to be essential for virus-mediated tumorigenesis and is therefore, an excellent MCC antigen for the generation of antitumor immune responses. As a foreign antigen, the LT oncoprotein avoids the obstacle of immune tolerance, which normally impedes the development of antitumor immunity. Ergo, it is an excellent target for anti-MCC immunotherapy. Since tumor-specific CD8+ T cells lead to better prognosis for MCC and numerous other cancers, we have generated a DNA vaccine that is capable of eliciting LT-specific CD8+ T cells. The DNA vaccine (pcDNA3-CRT/LT) encodes the LT antigen linked to a damage-associated molecular pattern, calreticulin (CRT), as it has been demonstrated that the linkage of CRT to antigens promotes the induction of antigen-specific CD8+ T cells.

**Results:**

The present study shows that DNA vaccine-induced generation of LT-specific CD8+ T cells is augmented by linking CRT to the LT antigen. This is relevant since the therapeutic effects of the pcDNA3-CRT/LT DNA vaccine is mediated by LT-specific CD8+ T cells. Mice vaccinated with the DNA vaccine produced demonstrably more LT-specific CD8+ T cells. The DNA vaccine was also able to confer LT-specific CD8+ T cell-mediated protective and therapeutic effects to prolong the survival of mice with LT-expressing tumors. In the interest of determining the LT epitope which most MCC-specific CD8+ T cells recognize, we identified the amino acid sequence of the immunodominant LT epitope as aa19-27 (IAPNCYGNI) and found that it is H-2k^b^-restricted.

**Conclusion:**

The results of this study can facilitate the development of other modes of MCC treatment such as peptide-based vaccines and adoptive transfer of LT-specific CD8+ T cells. Likewise, the MCC DNA vaccine has great potential for clinical translation as the immunologic specificity is high and the treatment strategy can be exported to address other virus-induced tumors.

## Introduction

While historically uncommon, merkel cell carcinoma (MCC) incidence has risen steadily over recent years [1,2]. MCC is a cutaneous neoplasm that originates from the mechanoreceptor merkel cells, which are derived from the embryonic neural crest. Highly malignant, this disease is prone to metastasis, and therefore afflicted patients have unfavorable prognosis and high mortality rates. MCC is among the most aggressive of skin cancers with patient mortality greater than that of melanoma [3]. Most often, MCC sufferers are seniors, immunosuppressed post-transplant patients, and immunodeficient individuals [4]; with the disease first appearing at sun-exposed areas such as the head and neck [5].

Investigation into the contributing factors that lead to MCC development has determined a possible infectious origin for the disease, specifically, the merkel cell polyomavirus (MCPyV) [6]. Studies throughout the world have shown that approximately 80% of MCCs contain MCPyV DNA [7]. It is unlikely that MCPyV is a passenger virus since tumor-derived MCPyV LT antigen contains unique mutations not found in wild-type virus. Furthermore, the viral genome is monocolonally integrated into the cell genome prior to tumor cell clonal expansion.

MCPyV is a small, oncogenic, virus with double-stranded circular DNA genome that is supercoiled. The viral genome contains a T antigen oncoprotein locus that can be expressed as four different alternatively spliced transcripts: the large T (LT) antigen, the two small T (ST) antigens, and the 57kT antigen [8]. It is likely that MCPyV is the etiologic agent responsible for the majority of MCC development, although approximately 20% of MCCs are MCPyV-negative. Furthermore, MCPyV infection is ubiquitous whereas the development of MCCs is unusual [9,10]. This has led to the speculation that MCPyV-positive MCCs entail specific conditions that enable viral-mediated oncogenesis.

The pathogenesis of MCPyV-positive MCC involves mechanisms that interfere with cell cycle checkpoints either through the disruption of regulatory sites during viral integration or through the inhibition of tumor suppressors. MCPyV DNA extracted from MCC cells has been found to contain mutations that cause the virus to be non-replicative. The LT antigen contains a premature stop codon that leads to the loss of helicase located at the C-terminus, which is necessary for viral replication [8]. The abolishment of viral replication ensures the infected cell is safe from lytic cycle-induced apoptosis [11]. Since the oncogenic N-terminus containing the retinoblastoma (RB) tumor suppressor protein-binding motif is preserved, the truncated LT antigen can instigate the transformation and proliferation of infected cells [11,12]. The mutational prerequisites that permit MCPyV-induced MCC may explain the frequency of disease development on the areas of the skin that are constantly exposed to UV.

In a previous study, we demonstrated that a DNA vaccine encoding a truncated LT antigen was able to produce antitumor effects against LT-expressing tumor cells. The efficacy of the codon-optimized DNA vaccine was found to be predominantly a result of CD4+ T cells [13]. However, we also determined that natural killer (NK) cells and CD8+ T cells have modest contributions to the therapeutic effects of the DNA vaccine [13]. Consequently, we decided to examine if the DNA vaccine encoding LT antigen could be tailored to favor the generation of LT-specific CD8+ T cells. Tumor-infiltrating CD8+ T cells are associated with improved prognosis and disease clearance for a variety of cancers [14] and have favorable prognostic value for the patient outcome [15]. For both virus-positive and –negative MCC, the presence of intratumoral CD8+ T cells is associated with improved outcome, reduced likelihood of metastasis and prolonged survival [16,17].

In the present study, we intended to identify the MHC class I-restricted immunodominant LT epitope and determine if the generation of LT-specific CD8+ T cells could be favored by linking calreticulin (CRT) with LT antigen in the DNA vaccine. CRT exposed on the cell surface of pre-apoptotic cells is a damage-associated molecular pattern (DAMP) recognized by dendritic cells as the green light for phagocytosis [18] and consequently is considered to be a marker of immunogenic cell death [19]. The inclusion of CRT in therapeutic DNA vaccines has been demonstrated to successfully promote the generation of antitumor immune responses in HPV-positive tumor models [20-22], justifying our rationale for the examination of anti-MCC immunotherapy with a DNA vaccine encoding CRT linked to the LT antigen.

## Materials and methods

### Mice

C57BL/6 mice were purchased from National Cancer Institute and maintained under specific pathogen-free conditions. All procedures were conducted in accordance with approved protocols and recommendations for the proper use and care of experimental animals.

### Peptides

49 overlapping peptides (20-mer, overlapping by 15 amino acids) spanning the MCPyV LT antigen (1-258aa) was synthesized by GeneScript Corportation (Piscataway, NJ) and used to identify the immunodominant MHC class I-restricted epitope as previously described [13]. All peptides used here were dissolved in 10% DMSO and stored at −20°C. The stock concentration and final concentration of peptides used were 20 μg/μl and 2 μg/μl respectively.

### Cell lines

The creation of B16F10 (B16/LT) cells expressing large T and GFP was achieved by previously described methods [13]. The C1R cell line missing most of its HLA class I alleles was derived from Epstein-Barr virus transformed B-cell line [23]. The C1R murine MHC class I transfectants, C1R/D^b^ and C1R/K^b^, were kindly provided by Michael Edidin (Johns Hopkins University, Baltimore, MD). All cells were maintained in RPMI medium supplemented with 2 mM glutamine, 1nM sodium pyruvate, 5 × 10^-5^ M β-mercaptoethanol, 100 IU penicillin/ml, 100 μg streptomycin/ml, and 10% fetal bovine serum.

### Plasmid DNA constructs and preparation

DNA vaccine pcDNA3-LT, encoding truncated large T antigen (aa 1–258) of Merkel cell polyomavirus (strain 350) [8], was made by cloning the large T antigen cDNA (1–774 nt) (GeneScript Corporation, Piscataway, NJ) into the EcoRI and HindIII sites of pcDNA3 (Invitrogen, Carlsbad, CA). To produce pcDNA3-CRT/LT, calreticulin (CRT) was removed from pcDNA3-CRT [24] with XbaI/EcoRI and cloned into pcDNA3-LT.

### Gene gun-mediated DNA vaccination

DNA vaccination with gene gun (Bio-Rad Laboratories, Berkeley, California) was performed as previously described [25]. Gold particles coated with pcDNA3, pcDNA3-LT, or pcDNA3-CRT/LT were delivered with a helium-driven gene gun to the shaved abdominal region of C57BL/6 mice (5 per group) at a discharge pressure of 400 psi. Mice were vaccinated, followed by two boosts at a 7-day interval with one of the DNA vaccines (2 μg). Splenocytes were harvested 1 week after the last vaccination.

### Intracellular cytokine staining and flow cytometry analysis

3.5 × 10^5^ of pooled splenocytes from each of the vaccinated groups were incubated overnight with 2 μg/ml LT peptide and 1 μg/ml GolgiPlug then treated with Cytofix/Cytoperm kit (BD Pharmingen, San Diego, California). Splenocytes were stained for surface CD8a and intracellular interferon-gamma (IFN-γ) using phycoerythrin (PE)-conjugated monoclonal rat anti-mouse CD8a (clone 53–6.7) and fluorescein isothiocyanate (FITC)-conjugated rat anti-mouse IFN-γ (BD Pharmingen) respectively. Flow cytometry was performed with FACSCalibur and analyzed by CELLQuest software (BD Biosciences, Mountain View, CA).

### Characterization of optimal LT peptide for LT-specific T cell activation

B cells (C1R, C1R/D^b^, and C1R/K^b^) were pulsed with 10 μg/μl of LT peptide (aa 19–27) at 37°C for 2 h then washed and irradiated at 10,000 rads. 5 × 10^6^ splenocytes mixed with 0.25 × 10^6^ target cells (E:T ratio 20:1) were seeded into 96-well round-bottom plates and incubated with Golgi plug for 5 h. Cells were washed once with FACScan buffer then stained for CD8a and IFN-γ as described above.

### In vivo tumor protection experiments

C57BL/6 mice (5 per group) were immunized with 2ug of pcDNA3, pcDNA3-LT or pcDNA3-CRT/LT by gene gun followed by two boosts at a 7-day interval. One week after the last vaccination, mice received subcutaneous B16/LT tumor challenge in the right flank (1 × 10^5^ cells/mouse). Tumor growth was monitored by inspection and palpation.

### In vivo tumor treatment experiments

C57BL/6 mice (5 per group) were subcutaneously inoculated with B16/LT tumor (1 × 10^5^ cells/mouse) in the right flank on D0. After 3 days of tumor growth, B16/LT-tumor bearing mice were intradermally administered 2ug of pcDNA3, pcDNA3-LT, or pcDNA3-CRT/LT by gene gun followed by two boosts at a 7-day interval. Mice were monitored for tumor growth by inspection and palpation. Tumor growth was measured twice a week starting from day 10 after tumor challenge.

### In vivo antibody depletion experiment

C57BL/6 mice (5 per group) were vaccinated by gene gun method with pcDNA3-CRT/LT DNA vaccine on D0. Vaccinated mice were boosted two times at the same dose and regimen at 1 week intervals. One day after last vaccination, mice were intraperitoneally injected with anti-CD8 every other day. Antibody-depleted mice were then challenged with B16/LT tumor (1 × 10^5^ cells/mouse) subcutaneously in the right flank on D22. Mice were monitored for evidence of tumor growth by inspection, palpation and tumor size was measured twice a week.

### Tumor size measurement

Tumor growth was monitored by visual inspection, palpation, and measured with Venire caliper twice a week as described previously. Tumor volumes were evaluated with the formula V (mm^3^) =3.14[largest diameter × (perpendicular diameter)^2^]/6.

### Statistical analysis

Statistical analysis was by the S.P.S.S. 17.0 program. All data are expressed as mean ± SD and are representative of at least two independent experiments. Comparisons between individual data points were made using a Student’s t-test. The values of *p* < 0.05 were considered significant.

## Results

### Identification of MHC class I-restricted immunodominant LT epitope

**Figure 1:**
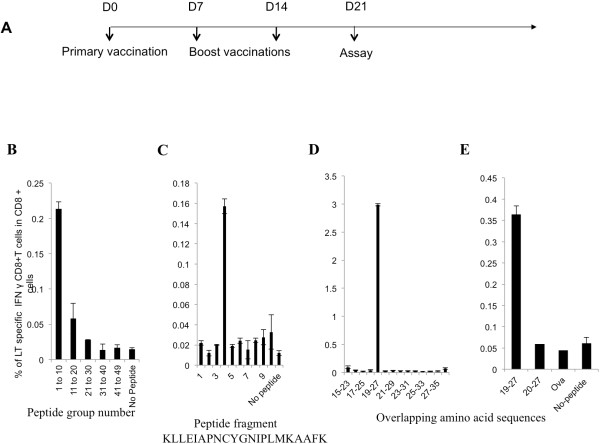
**Identification of MHC class I-restricted immunodominant LT epitope using overlapping peptides and splenocytes from mice vaccinated with pcDNA3-CRT/LT.** (**A**) Schematic diagram of vaccination schedule *in vivo*. C57BL/6 mice (5 mice/group) intradermally received pcDNA, pcDNA3-LT, or pcDNA3-CRT/LT 3 times by gene gun at a 7-day interval. Vaccination with pcDNA3-CRT/LT produced the most LT-specific CD8+ T cells therefore pooled splenocytes from pcDNA3-CRT/LT vaccinated mice were cultured *in vitro* with various overlapping LT peptides to find the immunodominant LT epitope. (**B**) Representative bar graph of flow cytometric data indicating the pool containing peptides #1-10 activated the most LT-specific CD8+ T cells. (**C**) Representative bar graph of flow cytometric data indicating that out of peptides #1 through 10, LT peptide #4 was able to activate the most LT-specific CD8+ T cells. (**D**) Representative bar graph of flow cytometric data suggesting amino acid 19–27 (IAPNCYGNI) of the LT antigen may be the immunodominant epitope determining the specificity of the vast majority of LT-specific CD8+ T cells. (**E**) Representative bar graph of flow cytometric data confirming that the suggested amino acid 19–27 (IAPNCYGNI) of the LT antigen may be the immunodominant epitope determining the specificity of the vast majority of LT-specific CD8+ T cells, and not the peptide occupying positions 20–27 (APNCYGNI). Ova (SIINFEKL) was used a negative control.

C57BL/6 mice were vaccinated with pcDNA3, pcDNA3-LT, or pcDNA3-CRT/LT. Splenocytes from vaccinated mice were stimulated with overlapping LT peptides and pcDNA3-CRT/LT was found to most effectively induce the production of LT-specific CD8+ T cells. Splenocytes from mice vaccinated with pcDNA3-CRT/LT were then stimulated with overlapping LT peptides to identify the immunodominant LT epitope. In order to map the immunodominant MHC class I-restricted LT epitope, we synthesized 49 overlapping 20-mer peptides (overlapping by 15 aa) spanning the MCPyV LT antigen aa 1–258. The overlapping peptides were sorted into a total of five pools, with four pools containing 10 peptides (#1-10, #11-20, #21-30, #31-40) and the fifth pool containing 9 peptides (#41-49). Each pool of peptides was individually incubated with splenocytes obtained from mice vaccinated with pcDNA3-CRT/LT DNA vaccine in the regimen depicted by the schematic diagram in Figure 1A. Stimulation with the pool containing peptides #1-10 led to the greatest amount of LT-specific CD8+ T cell response (Figure 1B), which suggested the immunodominant MHC class I-restricted LT epitope was located somewhere in the region spanned by peptides #1-10. The peptides from the pool containing peptides #1-10 were then separately incubated with splenocytes from mice vaccinated with pcDNA3-CRT/LT. As seen in Figure 1C, peptide #4 (aa 16–35) activated the greatest amount of LT-specific CD8+ T cells. To locate the immunodominant epitope within LT peptide fragment #4, 9-mer overlapping peptides (overlapping by 8 aa) spanning peptide #4 were individually incubated with splenocytes from vaccinated mice and as shown in Figure 1D, aa 19–27 (IAPNCYGNI) activated a significant amount of LT-specific CD8+ T cells. In order to define the probable immunodominant epitope more specifically, the LT-specific CD8+ T cell response generated by the aa 19–27 9-mer was compared to that of the 20–27 8-mer (APNCYGNI). Figure 1E indicates that the 9-mer generated a significantly greater number of LT-specific CD8+ T cells compared to the 8-mer overlapping peptide, aa 20–27. The results strongly suggest that the immunodominant LT epitope responsible for producing the vast majority of LT-specific CD8+ T cells is aa 19–27 (IAPNCYGNI).

### The MHC class I immunodominant LT epitope (aa 19–27) is H-2K^b^-restricted

**Figure 2:**
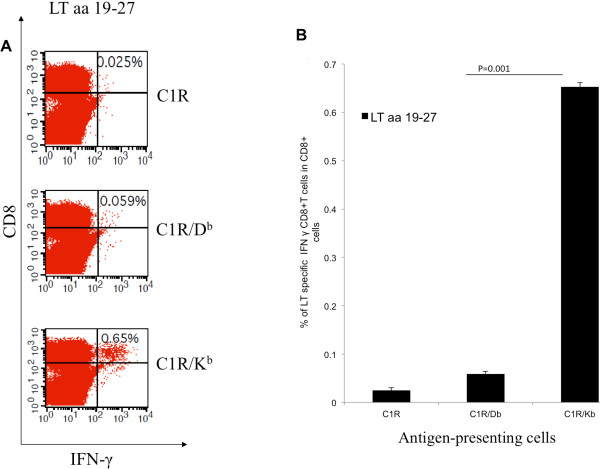
**MHC class I H-2**^**b **^**binding restriction of LT aa 19–27 epitope identified by intracellular cytokine staining and flow cytometry.** MHC class I binding restriction was determined by C1R transfectants, C1R/D^b^ and C1R/K^b^ , that were pulsed with immunodominant LT peptide (aa 19–27). (**A**) Representative flow cytometric data showing the amounts of LT-specific IFN-γ^+^ CD8^+^ T-cells after splenocytes from pcDNA3-CRT/LT vaccinated mice are stimulated by LT peptide-pulsed C1R, C1R/D^b^, or C1R/K^b^ cells *in vitro*. (**B**) Representative bar graph of flow cytometric data showing the proportions of LT-specific IFN-γ + CD8+ T cells per 3 × 10^5^ splenocytes following *in vitro* stimulation as described above. Note that LT epitope (aa 19–27) is H-2k^b^-restricted.

HLA-class I negative C1R antigen presenting cells were transfected to express H-2D^b^ and H-2K^b^ to produce C1R/D^b^ and C1R/K^b^ cells. The antigen presenting cells were pulsed with the immunodominant LT peptide (aa 19–27, IAPNCYGNI) identified by co-incubation of overlapping peptides with splenocytes from pcDNA3-CRT/LT immunized mice. Presentation of LT epitope (aa 19–27) by C1R/K^b^ led to the most efficient activation of LT-specific CD8^+^ T cells (C1R/D^b^ vs C1RK^b^; p < 0.001) whereas antigen presentation by C1R and C1R/D^b^ resulted in negligible T cell activation (Figure 2A). From this we expect the immunodominant LT epitope to be H-2k^b^-restricted (Figure 2B).

### DNA vaccine encoding CRT linked to LT significantly enhances the generation of LT-specific CD8^+^ T cells

**Figure 3:**
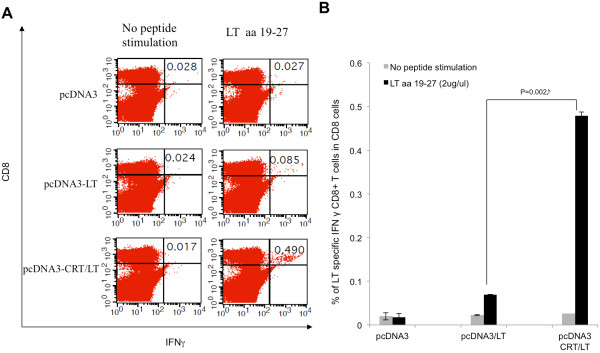
**pcDNA3-CRT/LT generates the most LT-specific (aa 19–27) CD8**^**+ **^**T cells.** C57BL/6 mice (5 mice/group) were immunized with DNA vaccines by gene gun in the same schedule as Figure 1A. Pooled splenocytes from mice vaccinated with pcDNA3 vector (control), pcDNA3-LT, and pcDNA3-CRT/LT were collected and cultured *in vitro* with either no peptide or amino acid 19–27 then stained for intracellular IFN-gamma and CD8^+^ T cell surface marker. (**A**) Representative flow cytometry dot plot of LT-specific CD8^+^ T cell activation after stimulation with amino acid sequence 19–27. (**B**) Representative bar graph of flow cytometric data.

We investigated whether the linkage of CRT to LT could augment the generation of LT-specific CD8+ T cells by vaccinating C57BL/6 mice with pcDNA3, pcDNA3-LT, and pcDNA3-CRT/LT. The splenocytes from vaccinated mice were stimulated with the prospective immunodominant LT epitope (aa 19–27). The mice vaccinated with pcDNA3-CRT/LT were found to produce the greatest number of CD8+ T cells that are specific for the LT epitope (aa 19–27) (Figure 3A), indicating the addition of CRT to LT greatly improves the efficacy of the DNA vaccine (p = 0.002) (Figure 3B). Moreover, the ability of the LT epitope (aa 19–27) to activate a large number of LT-specific CD8+ T cells further supports that aa 19–27 is the MHC class I-restricted immunodominant LT epitope.

### The linkage of CRT to LT enhances the protective effect of DNA vaccine

**Figure 4:**
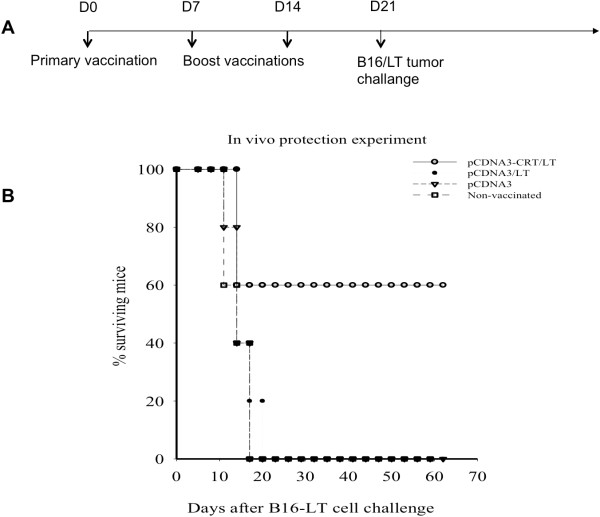
***In vivo *tumor protection experiment examining the antitumor effects generated by pcDNA3-CRT/LT against LT-expressing tumor.** (**A**) Schematic diagram of experiment schedule. (**B**) Survival curve of vaccinated mice subjected to tumor challenge. Note that vaccination with pcDNA3-CRT/LT greatly prolonged mouse survival after tumor challenge.

An *in vivo* tumor protection assay was conducted to determine the protective antitumor effects of pcDNA3-CRT/LT vaccine versus pcDNA3-LT vaccine. C57BL/6 mice were vaccinated with pcDNA3-CRT/LT, pcDNA3-LT, or empty pcDNA3 vector (control) followed by subcutaneous B16/LT tumor challenge (Figure 4A). Mice vaccinated with pcDNA3-CRT/LT had prolonged survival following tumor challenge in comparison to other groups (Figure 4B). Thus, our data show that pcDNA3-CRT/LT DNA vaccine can induce protective, immune-mediated antitumor effects.

### pcDNA3-CRT/LT DNA vaccine generates potent therapeutic antitumor effects against LT-expressing tumors in vaccinated mice

**Figure 5:**
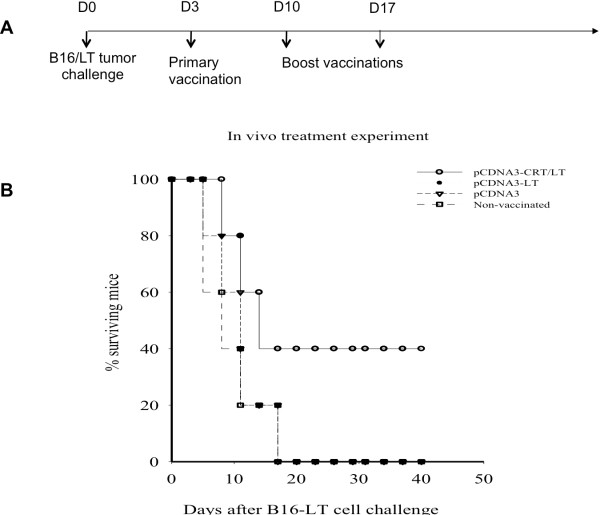
***In vivo *tumor treatment experiment with DNA vaccines.** (**A**) Schematic diagram of experiment schedule. (**B**) Survival curve of tumor-bearing mice treated with various DNA vaccines. Note that pcDNA3-CRT/LT had the greatest therapeutic effect and extended the survival of B16/LT tumor-bearing mice.

An *in vivo* tumor treatment experiment was conducted to determine the therapeutic effect of pcDNA3-CRT/LT DNA vaccine versus pcDNA3-LT vaccine. C57BL/6 mice challenged with B16/LT tumor cells were subsequently vaccinated with pcDNA3-CRT/LT, pcDNA3-LT or pcDNA3 (Figure 5A). Tumor-bearing mice vaccinated with pcDNA3-CRT/LT had the best survival (Figure 5B), suggesting that pcDNA3-CRT/L T DNA vaccine can produce therapeutic antitumor effects against LT-expressing tumors.

### CD8+ T cells play a role in the resulting antitumor effect after vaccination with pcDNA3-CRT/LT

**Figure 6:**
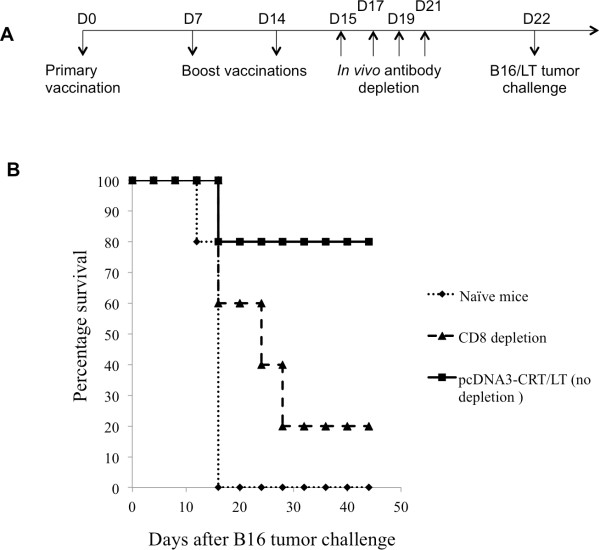
**The effect of CD8 T cells on tumor protection of the pCDNA3-CRT/LT vaccine**. (**A**) Schematic diagram of the vaccination regimen for *in vivo* antibody depletion experiments. C57BL/6 mice (5 per group) were vaccinated by gene gun with pcDNA3-CRT/LT DNA vaccine on D0. Vaccinated mice were boosted two times at the same dose and regimen at one week intervals. Beginning 1 day after last vaccination, vaccinated mice were intraperitoneally injected with anti-CD8 monoclonal antibody other day. Antibody-depleted mice were then challenged with B16/LT tumor (1 × 10^5^ cells/mouse) subcutaneously in the right flank on D22. Mice were monitored for evidence of tumor growth by inspection, palpation and tumor size was measured twice a week. (**B**) Survival analysis of B16/LT tumor-bearing mice treated with pcDNA3-CRT/LT DNA vaccine.

To demonstrate that CD8+ T cell depleted mice are no longer protected by the pcDNA3-CRT/LT vaccine, C57BL/6 mice were vaccinated with pcDNA3-CRT/LT, and an *in vivo* antibody depletion experiment was performed as indicated in Figure 6A. As shown in Figure 6B, 80% of the mice depleted of CD8+ T cells did not survive up to 45 days after tumor challenge compared to 20% of non-depleted mice. Therefore, depletion of CD8+ T cells led to loss of protective antitumor effects. These results indicate that the subset of CD8+ lymphocytes is important in mediating the antitumor effects generated by the pcDNA3-CRT/LT DNA vaccine.

## Discussion

In a previous study, we found that the pcDNA3-LT DNA vaccine had therapeutic effects that were predominantly mediated by LT-specific CD4+ T helper cells. Since tumor-reactive CD8+ T cells have consistently been found to be associated with improved patient outcomes, the present study examined whether the pcDNA3-LT DNA vaccine could be modified to favor the induction of LT-specific CD8+ T cells. We demonstrated that our pcDNA3-LT DNA vaccine encoding the LT oncoprotein (aa 1–258) from MCPyV can be modified to enhance the generation of LT-specific CD8+ T cells by linking CRT with the LT oncoprotein. The splenocytes obtained from mice vaccinated with the pcDNA3-CRT/LT vaccine contained significantly more LT-specific CD8+ T cells than the splenocytes obtained from mice vaccinated with pcDNA3-LT. Furthermore, splenocytes obtained from pcDNA3-CRT/LT -vaccinated mice were stimulated with LT oncoprotein overlapping peptides to identify aa 19–27 (IAPNCYGNI) as the immunodominant MHC class I-restricted epitope.

We then confirmed with peptide-pulsed HLA-transfected B cells (C1R) that the immunodominant LT epitope is H-2K^b^-restricted and leads to the greatest amount of CD8+ T cell activation (IFN- **γ)** in splenocytes obtained from pcDNA3-CRT/LT-vaccinated mice. A substantial amount of LT-specific CD8+ T cells were induced by vaccination with pcDNA3-CRT/LT DNA and the change in immune profile was effective at prolonging the survival of mice. The vaccine and the generation of LT-specific CD8+ T cell-mediated immunity were able to confer both protective and therapeutic effects against LT-expressing tumors.

The creation of the B16/LT tumor model is both necessary and important for the development of anti-MCC immunotherapy. The expression of MCPyV LT oncoprotein is speculated to be an obligatory aspect of the molecular mechanism behind the development of most MCCs [11,12]. As a result, this tumor model can potentially be used for future preclinical studies of MCC therapies. Most importantly, since the LT oncoprotein is foreign and does not have the issue of immune tolerance like most tumor-associated antigens, it is an ideal target for anti-MCC immunotherapy.

In the present study, we used CRT in our DNA vaccine to favor the development of LT-specific CD8+ T cells. However, there are numerous other agents that can be explored for the promotion of cell-mediated immunity. Previous DNA vaccine studies have identified additional factors that encourage the production of tumor-reactive CD8+ T cells. For example, the linkage of heat shock proteins, such as HSP70, to the human papillomavirus (HPV) oncoprotein was able to significantly increase the number of tumor-reactive CD8+ T cells [25]. An alternative strategy found to improve the MHC class I presentation of a tumor antigen is the linkage of gamma-tubulin to the HPV oncoprotein in a therapeutic DNA vaccine, which elicited a significant population of tumor-reactive CD8+ T cells in vaccinated mice with HPV + tumors [26]. Since a number of bacterial toxins, such as that of pseudomonas aeruginosa, have the ability to improve MHC class I presentation of an exogenous antigen by facilitating the translocation from endosomal/lysosomal compartments to the cytoplasm, the preferential generation of tumor-reactive CD8+ T cells can be achieved by linking the translocation domain of pseudomonas aeruginosa exotoxin A (ETA(dII)) to a tumor antigen [27].

The emphasis on the ability of an anti-MCC DNA vaccine to generate LT-specific CD8+ T cells stems from the fact that MCPyV-specific CD8+ T cells are consistently associated with improved patient outcomes [16,17]. Since MCPyV-specific CD8+ and CD4+ T cells are found in MCC patients [28], the research of LT-specific immunotherapy is a logical step in generating an effective treatment for virus-induced cancer. It is hypothesized that immune-mediated tumor regression is possible due to documented cases of spontaneous MCC regression [29,30]. Additionally, the importance of immunity in tumor clearance is emphasized by the significant representation of immunosuppressed individuals among MCC patients [5].

The results of the present study indicate that immunotherapy with a therapeutic MCPyV DNA vaccine is a compelling option for the treatment of MCPyV-positive MCC tumors. Due to the dependence on LT oncoprotein for MCPyV-mediated MCC development, the induction of LT-specific CD8+ T cells will lead to MCC-specific cytotoxicity. Given that the LT oncoprotein is foreign, it avoids the issue of immune tolerance. The identification of an MHC class I-restricted immunodominant epitope expands the possibilities for immunotherapy development. The clinical translation potential for this therapeutic MCC DNA vaccine is extremely high as the strategy can be exported to address other virus-induced tumors. Moreover, the efficacy of the DNA vaccine can be optimized to generate LT-specific CD8+ and/or CD4+ T cells.

## Competing interests

The authors declare they have no competing interests.

## Authors' contributions

BG and SP performed animal experiments. LH performed immunological assays. CW and CH prepared the manuscript. RV, TW, and CH conceived and designed the study. All authors read and approved the final manuscript.
